# Shared first authorship

**DOI:** 10.5195/jmla.2019.700

**Published:** 2019-10-01

**Authors:** Amy Lapidow, Paige Scudder

**Affiliations:** Research and Instruction/Circulation Librarian, Hirsh Health Sciences Library, Tufts University, Boston, MA 02111, amy.lapidow@tufts.edu; Research and Education Librarian, Biomedical Libraries, Dartmouth College, Hanover, NH 03755, paige.n.scudder@dartmouth.edu

## Abstract

In most scientific communities, the order of author names on a publication serves to assign credit and responsibility. Unless authors are presented in alphabetical order, it is assumed that the first author contributes the most and the last author is the driving force, both intellectually and financially, behind the research. Many, but not all, journals individually delineate what it means to be a contributing author and the nature of each author’s role. But what does this mean when a paper has co-first authors? How are academic librarians going to handle questions surrounding co-first authorship in an era in which author metrics are important for career advancement and tenure? In this commentary, the authors look at the growing trend of co-first authorship and what this means for database searchers.

In 1981, the largest number of authors on any paper indexed by Clarivate Analytics was 118. In 2006, this number was 2,500, which was quickly topped in 2008 with 3,000 authors [1]. Currently, a physics paper with 5,154 authors holds the record for the largest number of contributors on a single paper. As the pressure to publish, specialization of research, wider collaborative efforts, team-based research, and honorary authorship continue to increase, the number of authors per article has been rising across scientific fields. As authorship in academia contributes to hiring and promotion processes [2], these increases have resulted in many disciplines and journals establishing rules to determine who qualifies for academic authorship [3]. Furthermore, there is a current trend toward denoting the nature of the contribution of each author. No longer can the role or status of authors be determined solely by the position of their names in the author byline.

Many journals follow guidelines set by the International Committee of Medical Journal Editors (ICMJE), which created a list of recommendations to clarify the definition and responsibilities of an author [4]. Despite these definitions, no criteria have been outlined for defining first author, nor have any recommendations been made in regard to author order. By tradition in medical literature, if not listed alphabetically, the first author makes the largest contribution and the last author is the most senior or principal investigator [5]. Despite this tradition, there are no firm guidelines in place to ensure or guarantee a fair interpretation of authors’ contributions [6].

As multicenter studies and multiauthor collaborative research and publication grow, the role of the traditional first author is fading. This is problematic in that authorship and author order are used to determine academic achievement for the purposes of promotion, allocation of research time, and funding [6]. In 1999, *Nature* adopted a policy of transparency, publishing each author’s role in the research and article preparation [7], and other journals have since followed suit. However, reporting of authors’ roles—if it takes place at all—and whether or not the reporting coincides with the ICMJE’s guidelines depends on the journal. Articles published in *JAMA* are most likely to fulfill ICMJE criteria for authorship, whereas middle authors in the *Lancet* are least likely [5]. Renowned scientific publishers, such as Elsevier and Springer, allow the article’s contributors to decide the proper order of authors during the publishing process [6]. Despite the growing number of guidelines, there are no recommendations in place to guide a paper that has been written by coauthors who have made equal contributions to the publication.

Shared co-first authorship is defined as two or more authors who have worked together on a publication and contributed equally [8]. This equal contribution is often indicated in the fine print of a published paper or in an investigator’s curriculum vitae [9]. Some journals publish articles in which shared coauthorship is described, making it easy to determine author contribution. For instance, *Gastroenterology* acknowledges up to two co-first authors by bolding their names in the reference section but not in the body of the manuscript [10]. While suggestions have been made to make equal authorship more findable in databases, in-text citations, and bibliographies, no unified system has been created [11]. How, then, can readers determine the roles of authors when looking at their order in a citation or bibliographic record?

If an article has more than two authors, do we assume that the second author is a co-first author or that the last author is? How can we retrieve citations containing co-first authors? How are health sciences librarians to handle these questions? The authors suggest that some sort of searchable field needs to be instituted, perhaps something akin to personal name as a subject or the creation of a coauthorship Medical Subject Headings (MeSH) term in MEDLINE.

In 2011, Sandra Schmid, president of the American Society of Cell Biology, asked administrators of PubMed to consider this problem. An American Society of Cell Biology team searched PubMed Central and could only find 0.8% of 10,000 articles that identified a co-first author, a statistic that should have placed a greater emphasis on the problem [7]. There are approximately 3 million articles in PubMed Central and 26 million articles in PubMed. As research continues to be conducted and published, it can be expected that these numbers will continue to grow. If 99.2% of 10,000 articles with co-first authors in 2011 could not be found by searchers and the number of co-first authors has increased since then, one is left to wonder how many articles a similar search would miss today. Perhaps it is time to rerun the search to demonstrate that its continued difficulty would illustrate the lack of delineation of co-first authors in the major bibliographic databases.

Why do health sciences librarians need to be able to identify articles with co-first authors in databases? Because our institutions’ authors are being asked to detail their individual roles on manuscripts, leading most people to run a search in a standard bibliographical database. Librarians are increasingly being asked to guide patrons though this process or to conduct the search on their behalf. If the database cannot support this search, then the author or librarian has to look at each paper to determine if there is any indication of author role—a time-consuming task in this day of systematic reviews, long bibliographies, and hyperauthorship.

Additionally, whether author acknowledgment will affect an author’s metrics needs to be considered. As of now, most metrics are based on an author’s name appearing on a manuscript, which means that the same measure of credit is applied to every author on a publication [5]. Whether one uses times cited, h-index, Publons, essential science indicators, or any other metric, they all use appearance of a name, not the weight of contribution.

Perhaps it is time to encourage a weighting system to properly delineate author metrics. For example, the normalized h-index offers a way to “even the playing field” by dividing the original index by the total number of published papers, and the trend h-index takes into account the “age” of a citation [12]. Other h-index variants specifically address author order or rank articles by the number of authors in the author byline [6, 13]. It is time to seriously consider which metrics or formulae provide the best reflection of those publishing.

Many journals skirt the issue by proclaiming that this is an emerging trend. However, as early as 1983, authors in the *British Medical Journal* called for defining the roles of authors as the magnitudes of their contributions might not be equal or obvious [14]. Co-first authorship is a growing trend that is likely to continue over time; especially given the time constraints, specialization of expertise, and competition among today’s scholarly researchers. Co-first authorship is predicted to level off at 20%–40%, depending on the discipline [9].

It is time to look at the various blueprints that have been put forth and create a single set of guidelines that can be used to assign value to authorship across disciplines, perhaps by the ICMJE. It is also time to take another look at how papers with co-first authors are indexed so that they can be found and identified by searchers.

Librarians should facilitate these conversations. Can we petition the National Library of Medicine or other standard databases to create an indexed field? How can we make our concerns heard by journals to standardize their practices in weighting author contributions? This problem is not going to go away, especially when so many people are starting to view coauthorship as a way to mentor peers across the field, to build relationships with those who hold similar interests and concerns, and to pool institutional resources and professional networks [15] and build reputation and legacy [6]. Librarians have the opportunity and responsibility to ensure that searchers can find co-first authors, starting with discussing authorship and author order when researchers come in with publishing questions, continuing with discussions on how to identify co-first authors in a research paper, and ending with the articles being findable in databases. After all, if librarians cannot look at a citation and determine whether or not an author is a co-first author, can anyone?

**Amy Lapidow**, amy.lapidow@tufts.edu, Research and Instruction/Circulation Librarian, Hirsh Health Sciences Library, Tufts University, Boston, MA 02111

**Paige Scudder**, paige.n.scudder@dartmouth.edu, Research and Education Librarian, Biomedical Libraries, Dartmouth College, Hanover, NH 03755
